# Experimental Resin-Based Monoblock Endodontic Obturation System

**DOI:** 10.1155/2019/3512606

**Published:** 2019-11-07

**Authors:** Thiago Machado Pereira, Evandro Piva, Carlos Enrique Cuevas-Suárez, Luiz Evaristo Ricci Volpato, Manoel dos Santos da Silva Neto, Kellin Pivatto, Jesus Djalma Pécora, Álvaro Henrique Borges

**Affiliations:** ^1^University of Cuiaba, Cuiaba, Brazil; ^2^Federal University of Pelotas, Pelotas, Brazil; ^3^Autonomous University of Hidalgo State, Pachuca, Mexico; ^4^University of São Paulo, São Paulo, Brazil

## Abstract

**Objective:**

The aim of the present study was to characterize a novel resin-based monoblock endodontic obturation system consisting of a polymeric cone and a resin-based endodontic sealer.

**Methods:**

The preliminary tests performed for the experimental cone were as follows: cohesive strength, dimensional stability, standardization of the diameter and taper, calcium ion release, and radiopacity, for the characterization of the experimental sealer, film thickness and flow tests were performed. Tests were performed according to the American National Standards Institute/American Dental Association (ANSI/ADA) Standards Nos. 57 and 78. The experimental cone was compared to gutta-percha, whereas the experimental sealer was compared to AH Plus. Data were analyzed by Student's *t*-test (*α* = 0.05).

**Results:**

The experimental cone had superior values for cohesive strength and dimensional stability compared to gutta-percha. Regarding calcium ion release, the experimental system continued to release calcium ions after 30 days. Film thickness was similar for both endodontic sealers; conversely, the experimental resin-based sealer achieved higher values for flow compared to AH Plus.

**Conclusion:**

The experimental resin-based monoblock obturation system fully met the requirements of the ANSI/ADA Standard No. 78 and the ANSI/ADA Standard No. 57, except for radiopacity. Considering this, further studies are still needed to evaluate other radiopacifiers and the effect of their incorporation on the physicochemical properties of this novel resin-based monoblock endodontic obturation system.

## 1. Introduction

The ultimate goal in endodontics is to eliminate the etiology of pulpal and periradicular disease through the disinfection and obturation of the root canal system [1]. The obturation should prevent bacteria and tissue fluids from reentering the root canal space, with a material that is dimensionally stable and biocompatible [2]. Several techniques and materials have been developed over the last decades aiming to achieve the optimal properties required for the root canal filling process. Hence, many studies have compared the performance of obturation techniques in different aspects; however, they have yielded disparate results [2–4]. To date, no filling material or technique has been considered ideal, and the occurrence of failure has been observed when trying to obtain a void-free homogeneous sealing of root canals [2].

Nowadays, gutta-percha is still the most commonly used material for endodontic obturation [5]. As gutta-percha does not bind by itself to the root canal walls, it must be used in conjunction with sealers in order to optimize the sealing process [6, 7]. Although predictable clinical results have been reported with the use of gutta-percha and endodontic sealers by the application of different techniques [2], there is interest in the use of adhesive concepts in endodontics [8]. Furthermore, the introduction of endodontic instrumentation systems that simulate the geometry of the main cones makes the investigation of single-cone obturation techniques an important prospect [3, 9].

Monoblock obturation can be performed with the use of a single-cone technique associated with a sealer, without the need for further technical resources, providing adhesion both at the interface between the sealer and the dentinal tubules and at the interface between the sealer and the obturating cones [9–11]. In order to improve the attachment of monoblock obturation, resin-based filling materials have been tested with promising results [12–14]. Thus, the aim of the present study was to develop and characterize a resin-based monoblock obturation system. The null hypothesis tested was that the experimental obturation system would achieve a performance similar to that of gutta-percha and AH Plus sealer.

## 2. Materials and Methods

### 2.1. Formulation of the Materials

The resin-based monoblock obturation system developed in this study consisted of a resin-based cone combined with a dual-cure resin-based sealer. The experimental resin-based cones were compared to gutta-percha pellets (Obtura Spartan, Algonquin, IL, USA), and the experimental sealer was compared to AH Plus (Dentsply Sirona, York, PA, USA). The compositions of the experimental materials are summarized in Table 1.

## 3. Characterization of the Experimental Cone

### 3.1. Cohesive Strength

Dumbbell specimens (10.0 mm length, 2.0 mm width, and 1.0 mm thickness) were produced using a metal mold (*n* = 10). The resin-based material formulated for the experimental cone was placed into the metal molds and light cured for 30 s on both the top and bottom surfaces. For the gutta-percha group, pellets were heated with a lamp until plastification occurred, and the molds were filled. The specimens were dry stored for 24 h at room temperature, and the area of the constriction zone was measured with a digital caliper (Mitutoyo, Suzano, SP, Brazil). The specimens were then attached to a metal device with cyanoacrylate-based glue and tested for ultimate tensile strength in a universal testing machine (DL 500; EMIC, São José dos Pinhais, PR, Brazil) at a crosshead speed of 0.5 mm/min until fracture. The cohesive strength was calculated and expressed in MPa.

### 3.2. Dimensional Stability

Cylindrical specimens of each material were fabricated using metal molds (6.0 mm length and 4.0 mm diameter) (*n* = 8). After preparation, the length of each specimen was measured with a digital caliper (Mitutoyo, Suzano, SP, Brazil). The specimens were then placed in Eppendorf tubes containing 2 mL of distilled water and stored at 37°C for 30 days, when they were measured again. The percentage of dimensional change (DC) was calculated by the following formula:

(1) $$\begin{array}{c} \text{DC}=\frac{L30–L}{L}\times 100, \end{array}$$

where *L* is the initial length of the specimen (mm) and L30, the length after 30 days of storage.

### 3.3. Calcium Ion Release

Cylindrical specimens (5 mm diameter and 1 mm thickness) were fabricated using a metal mold (*n* = 3). Specimens were placed at the bottom of cylindrical polystyrene containers with 10 mL of ultrapure water and stored at 37°C. After 3 h, 24 h, 72 h, 168 h, and 720 h, the water was collected and renewed. After collecting the water, 0.2 mL of an ionic strength adjuster (HI4004-00; HANNA instruments, Póvoa de Varzim, Portugal) was added to the solution. Measurements were made, in mV, using a calcium ion selective probe (HI 4104; HANNA instruments, Póvoa de Varzim, Portugal) connected to an ion reader (HI 5222; HANNA instruments, Póvoa de Varzim, Portugal). To calculate the concentration of calcium ions in ppm, a calibration curve (mV × ppm) was constructed by diluting the 0.1 M calcium standard solution (HI 4004-01; HANNA instruments, Póvoa de Varzim, Portugal) at the concentrations of 1, 5, 25, 50, 75, and 100 ppm.

### 3.4. Standardization of Diameter and Taper

Experimental cones were fabricated by molding gutta-percha points (Dentsply Sirona, USA) with a transparent fluid addition silicone (Scan Translux; Yller, Brazil). Impressions were then filled with a resin blend (Table 1) and photoactivated for 2 minutes using a photopolimerization unit with irradiance or 950 mw/cm^2^ measured by using a radiometer (Ultra Radii, SDI, Australia). After light curing, experimental cones were demolded for the impression and stored under humidity-free conditions until their use. A total of 60 experimental cones were manufactured according to the following specifications: IS0 02 #35 (*n* = 10); IS0 02 #40 (*n* = 10); IS0 02 #50 (*n* = 10); IS0 02 #60 (*n* = 10); IS0 02 #70 (*n* = 10); and IS0 02 #80 (*n* = 10). The American National Standards Institute/American Dental Association (ANSI/ADA) Standard No. 78 [15] was used as a reference for comparing the diameters, where a tolerance level of ±0.07 mm for polymeric cones is established. Each cone was placed on a millimeter ruler (Dentsply Sirona, York, PA, USA) so that the least calibrated tip exactly matched the zero mark of the ruler, with the remainder of the cone lying along the increasing markings of the ruler. A digital caliper (EDA, São Paulo, SP, Brazil) was used to measure the cone diameter at the least calibrated tip (D0), and the values were recorded. To verify the taper, the diameter at the 16 mm recess length (D16), determined from the least calibrated tip to the most calibrated tip of the cone, was measured and then compared to the ANSI/ADA values, considering the tolerance level of ±0.07 mm. All values were arranged in tables, where the means and standard deviations of the obtained measurements were calculated.

### 3.5. Radiopacity

A bipartite metal matrix measuring 5 mm in inner diameter and 1 mm in thickness (*n* = 5) was used to prepare the specimens of the experimental resin-based material and gutta-percha. Specimens from each group were placed on an occlusal phosphor plate (VistaScan Plus; Dürr Dental AG, Bietigheim-Bissingen, Germany) and radiographed with an X-ray apparatus (Ion 70x; Procion, Ribeirão Preto, SP, Brazil) at 70 kVp, 8 mA, an exposure time of 0.2 s, and a focal length of 40 cm. An aluminum step wedge measuring 50 × 20 mm with varying step thickness (every 1 mm) was used as a reference. A total of three radiographs were taken for each group and processed with VistaScan Plus (DBSWIN Imaging Software; Dürr Dental AG, Bietigheim-Bissingen, Germany). The images were captured at 300 dpi and 8-bit mode and stored in JPEG format. The digital images were analyzed with Adobe Photoshop CC 2015 (Adobe Systems, San José, CA, USA) using the histogram tool. A standardized sized circle was drawn in the center of each sample, and the average of the grayscale values of the pixels in the selected area was calculated and recorded.

## 4. Characterization of the Experimental Sealer

### 4.1. Film Thickness

Film thickness was evaluated according to the ANSI/ADA Standard No. 57 [16]. Two 5 mm-thick glass plates measuring 40 × 40 mm in length and having a contact area of 200 ± 25 mm^2^ were used. The thickness of the two overlapping glass plates was measured with a digital caliper (EDA, São Paulo, SP, Brazil), and the area of measurement (center of the plate) was marked with a pen. Sealers were spatulated and 0.05 mL of the sealer was placed in the center of one of the plates. After 180 ± 10 s, the other plate was placed on top of the sealer and a vertical load of 150 N was applied to the top surface of the plates. After 10 min, the thickness of the two overlapping plates with the sealer between them was measured with a digital caliper (EDA, São Paulo, SP, Brazil). The film thickness of each sealer was calculated as the difference between the thickness of the plates with and without the sealer. The test was performed in triplicate for the two sealers evaluated (*n* = 3).

### 4.2. Flowability

Flow was determined according to the ANSI/ADA Standard No. 57. A total of 0.05 mL of the manipulated material was dispensed on a glass plate measuring 40 × 40 × 5 mm. After 180 s from the start of mixing, another glass plate of the same dimensions and a 100 g weight was placed on top of the mixture. After 10 min, the weight was removed. The largest and smallest diameters of the compressed material were measured using a digital caliper (EDA, São Paulo, SP, Brazil). The test was performed in triplicate for each sealer.

### 4.3. Statistical Analysis

Preliminary statistical tests were performed to verify the normality of data distribution using SigmaPlot, version 12.0 (Systat Inc., San José, CA, USA). Data were then analyzed by Student's *t*-test. The level of significance was set at *α* = 0.05 for all analyses.

## 5. Results

The resin-based material formulated for the experimental cone achieved significantly higher values for cohesive strength and dimensional stability than gutta-percha (*p* < 0.05) (Table 2). Regarding calcium ion release, the experimental material released calcium ions until 30 days. For radiopacity, the experimental cone showed values of 1.63 (0.47) mm, which were statistically significant lower than the gutta-percha values. With regards to the analysis of the experimental resin-based sealer, the flow analysis showed higher values for AH Plus. On the other hand, film thickness was not significantly different between the experimental sealer and AH Plus (Table 2).

Table 3 shows the reference values of the ANSI/ADA Standard No. 78 for resin-based cones and the mean and standard deviation values, in mm, of the diameters at the tip (D0) and at 16 mm from the tip (D16) of the experimental cones. Regarding diameter and taper analysis, experimental resin-based cones fully conformed to the requirements of the ANSI/ADA Standard No. 78.

## 6. Discussion

In this study, an endodontic resin-based monoblock obturation system consisting of a polymeric flexible cone and a dual-cure resin-based sealer was developed and characterized. The properties of the components of the experimental obturation system were compared with gutta-percha and an epoxy-based commercial sealer. According to the results, most of the properties evaluated were statistically significantly different. Considering this, the null hypothesis tested was partially rejected.

The concept of composite resin in dentistry, introduced in the 1950s, has been slowly accepted by the scientific community. Although initial trials were strictly aimed at preventive and restorative dentistry, orthodontics and endodontics have now adopted this concept [5, 17]. One aspect that contributes to the development of resin-based monoblock obturation systems is the understanding that gutta-percha does not bind to dentin or to sealers [5, 8, 18, 19]. Conventional materials are being used with relative success, but recent advances in adhesive technology have led to the investigation of new options of endodontic sealers and cones based on adhesive properties and polymer resin technology [8, 20]. Therefore, the novelty of this experimental material in relation to other systems available in the market is the possibility of filling in a single unit associated with the release of calcium ions. Additionally, this resin-based experimental system is intended to be used together with an adhesive system, which could help to bind to the dentin, improving the sealing properties of the material.

ANSI/ADA establishes the standards and tests to be used in the development of these novel materials and in the evaluation of their physicochemical properties in order to promote greater scientific rigor. The ANSI/ADA Standard No. 78 used for characterization of the obturating cone was revised in 2013 and includes the following tests: diameter and taper, radiopacity, resistance, and visual inspection where the cones should be rounded and present smooth surface and uniform appearance. The ANSI/ADA Standard No. 57 used for characterization of the sealer was revised in 2000 and includes the following tests: flowability, film thickness, working time, setting time, solubility and disintegration, radiopacity, and dimensional stability. Therefore, these two standards were used in the present study with the purpose of developing and characterizing a dual-cured resin-based monoblock endodontic obturation system. The experimental cones were manufactured from the experimental resin-based material and light cured simulating the gutta-percha cones. The experimental sealer was manufactured from a similar formulation, but it was subjected to both chemical activation and light activation. Different radiopacifiers were used for the formulation of each paste. This strategy was used to achieve materials with different coloration to ensure a homogeneous mixture.

The ANSI/ADA Standard No. 78 recommends performing fragility tests in obturating cones. According to the cohesive strength testing, the experimental material showed higher values for cohesive strength than gutta-percha. The greater strength of the experimental material may be related to its ability to be removed as a single unit during endodontic retreatment. However, there is still no evidence of improved ability to remove resin-based obturation systems compared to gutta-percha, since no system is able to eliminate all obturation material remnants during retreatment [21, 22].

The dimensional change analysis yielded higher values for the experimental cone than for the gutta-percha material. Didato et al. [23] reported that, when exposed to water, the expansion of a hydrophilic obturating cone increases significantly. This expansion may result in a reduction of voids within the obturation material. Also, this expansion could reduce the possibility of marginal infiltration, but it may also result in positive root strains and possible root fractures. However, none of these hypotheses can be proven by the methodologies applied here. Testing syneresis and water sorption of this novel material is indicated for future studies to accurately assess the three-dimensional change.

With regards of the calcium ions release, the experimental material showed calcium ion release until 30 days of immersion, representing a major advantage of this material. The release of calcium ions is related to the presence of MTA in the formulation of the experimental cone. Calcium release of MTA-based materials occurs during the setting reaction, which results in the formation of calcium hydroxide, which subsequently dissociates into calcium and hydroxyl ions. Such ions are responsible for the antimicrobial effects and biological activity observed in this type of materials [20].

With regards to the radiopacity, the radiographic densities obtained, in grayscale values, provided the average of the radiographic density, in Al mm, of each material. According to the ANSI/ADA Standard No. 57, all root canal obturation materials must have a radiopacity greater than the equivalent of 3 mm of aluminum. The results showed that the radiopacity of the experimental material was lower than minimum established by ANSI/ADA, therefore requiring the evaluation of other radiopacifiers in future studies, as well as their effect on physicochemical properties.

When assessing the standardization of cone diameter and taper, all tested specimens met the requirements of the ANSI/ADA Standard No. 78. A possible explanation is the high tolerance level determined by ANSI/ADA. Thus, even when following the recommended guidelines, some variability should be expected within the dimensions of different cones [24]. The use of a calibrator or endodontic ruler is therefore indicated.

The method used for flow testing, following the recommendations of the ANSI/ADA Standard No. 57, was flatness or extensibility, by calculating the average area obtained when the sealer is subjected to a constant load for a given time. The ANSI/ADA Standard No. 57 determines that sealers should not have a flow-through diameter of less than 20 mm, and the experimental sealer was in accordance with this specification. The flowability of a sealer is an important factor for the clinical performance of the material, as it interferes with the ability to penetrate small irregularities in the dentin and lateral canals, giving the resin-based sealer greater adhesive capacity [25]. Regarding film thickness, the experimental material presented values similar to those of AH Plus. The experimental material met the ANSI/ADA manufacturing specifications which determine that sealers should not have a film thickness greater than 0.05 mm. Film thickness is a specific characteristic of each sealer, since it is related to factors such as shape and size of the particles, consistency, and degree of polymerization. It should be noted that the amount of particles of a resin-based sealer is also related to its mechanical properties, where the greater the film thickness, the better the mechanical properties and the lower the polymerization contraction [26].

The preliminary test results of our resin-based monoblock obturation system are promising, but the analysis of other physicochemical properties and biological properties is still necessary. Although the concept of creating homogeneous units within the root dentin is excellent in theory, evaluating the performance of monoblock obturation is challenging [10, 27]. Therefore, further studies are warranted to evaluate other radiopacifiers and the effect of their incorporation on the physicochemical properties of this resin-based monoblock endodontic obturation system.

## 7. Conclusion

Based on the present results, it is possible to conclude that the experimental resin-based monoblock obturation system fully conforms to the requirements of the ANSI/ADA Standard No. 78 and the ANSI/ADA Standard No. 57, except for radiopacity. A major advantage is the presence of calcium ion release after 30 days, which could provide the material antibacterial properties and bioactivity.

## Figures and Tables

**Table 1:** Chemical composition of the tested endodontic sealers and cones.

Material	Composition
*Endodontic cone*	
Experimental cone	Exothane 32, UDMA, BisEMA 30, CQ, EDAB, MTA
Gutta-percha cone	Gutta-percha, zinc oxide, heavy metal sulfates, waxes

*Endodontic sealer*	
Experimental sealer paste A	Exothane 32, DHPTE, BiO_2,_ SiO_2_
Experimental sealer paste B	UDMA, BisEMA 30, BPO, Ta_2_O_5_, SiO_2_
AH Plus sealer paste A	Bisphenol-A-diglycidyl-ether, calcium tungstate, zirconium oxide, aerosol, iron oxide
AH Plus sealer paste B	Amino-1-adamantane, N,N-dibenzyl-5-oxanonandiamine-1,9, TCD-diamine, calcium tungstate, zirconium oxide, silicon oxide

UDMA: urethane dimethacrylate (Esstech Inc. Essington, PA, USA); CQ: camphorquinone (Esstech Inc. Essington, PA, USA); EDAB: ethyl-4-dimethylaminobenzoate (Sigma-Aldrich Chemical Co. Milwaukee, WI, USA); DHPTE: N,N-bis(2-hydroxyethyl)-p-toluidine (Sigma-Aldrich Chemical Co. Milwaukee, WI, USA); BPO: benzoyl peroxide (Sigma-Aldrich Chemical Co. Milwaukee, WI, USA); SiO_2_: silicon dioxide (Evonik, Essen, Germany); Ta_2_O_5_: tantalum(V) oxide (Sigma-Aldrich Chemical Co. Milwaukee, WI, USA); BiO_2_: bismuth oxide (Sigma-Aldrich Chemical Co. Milwaukee, WI, USA). MTA: aggregate trioxide mineral (MTA Angelus White, Angelus, PA, Brazil).

**Table 2:** Physicochemical properties of the tested endodontic sealers and cones.

Properties	Materials	
*Cone*	Gutta-percha	Resin-based
Cohesive strength (MPa)	4.979 ± 1.50^a^	6.606 ± 1.62^b^
Dimensional change (%)	0.355 ± 0.15^a^	1.555 ± 0.57^b^
Calcium ion release (ppm)	<1^a^	2.524 ± 0.30^b^
Radiopacity	8.19 ± 0.28^b^	1.63 ± 0.47^a^

*Sealer*	AH Plus	Resin-based
Film thickness (mm)	0.01^a^	0.01^a^
Flow (mm)	16.53 ± 0.36^a^	13.77 ± 0.24^b^

Data followed by different letters are statistically different in the same row (*p* < 0.05).

**Table 3:** Cone diameters, in mm, at D0 and D16.

#	35	40	50	60	70	80
ANSI/ADA D0 reference values	0.35 ± 0.07^a^	0.40 ± 0.07^a^	0.50 ± 0.07^a^	0.60 ± 0.07^a^	0.70 ± 0.07^a^	0.80 ± 0.07^a^
Experimental cone D0	0.36 ± 0.01^a^	0.40 ± 0.01^a^	0.49 ± 0.02^a^	0.61 ± 0.02^a^	0.68 ± 0.03^a^	0.81 ± 0.03^a^
ANSI/ADA D16 reference values	0.67 ± 0.07^b^	0.72 ± 0.07^b^	0.82 ± 0.07^b^	0.92 ± 0.07^b^	1.02 ± 0.07^b^	1.12 ± 0.07^b^
Experimental cone D16	0.66 ± 0.01^b^	0.71 ± 0.03^b^	0.80 ± 0.03^b^	0.89 ± 0.03^b^	1.05 ± 0.04^b^	1.15 ± 0.04^b^

D0: diameter at the least-calibrated tip of the cone; D16: diameter at the 16 mm recess length; ANSI/ADA: American National Standards Institute/American Dental Association. Data followed by different letters are statistically different in the same row (*p* < 0.05).

## Data Availability

Data sharing is not applicable in this study as no new datasets were generated in the research.
